# Association between parasite microbiomes and caste development and colony structure in a social trematode

**DOI:** 10.1111/mec.16671

**Published:** 2022-09-02

**Authors:** Fátima Jorge, Nolwenn M. Dheilly, Céline Froissard, Robert Poulin

**Affiliations:** ^1^ Otago Micro and Nano Imaging, Electron Microscopy Unit University of Otago Dunedin New Zealand; ^2^ School of Marine and Atmospheric Sciences Stony Brook University Stony Brook New York USA; ^3^ Unité Génétique Virale de Biosécurité, Agence Nationale de Sécurité Sanitaire de l'Alimentation, de l'Environnement et du Travail ‐ Laboratoire de Ploufragan‐Plouzané ANSES Ploufragan France; ^4^ Department of Zoology University of Otago Dunedin New Zealand

**Keywords:** castes, division of labour, microbiome, parasite, sociality, symbiont

## Abstract

Division of labour through the formation of morphologically and functionally distinct castes is a recurring theme in the evolution of animal sociality. The mechanisms driving the differentiation of individuals into distinct castes remain poorly understood, especially for animals forming clonal colonies. We test the association between microbiomes and caste formation within the social trematode *Philophthalmus attenuatus*, using a metabarcoding approach targeting the bacterial 16S SSU rRNA gene. Clonal colonies of this trematode within snail hosts comprise large reproductive individuals which produce dispersal stages, and small, non‐reproducing soldiers which defend the colony against invaders. In colonies extracted directly from field‐collected snails, reproductives harboured more diverse bacterial communities than soldiers, and reproductives and soldiers harboured distinct bacterial communities, at all taxonomic levels considered. No single bacterial taxon showed high enough prevalence in either soldiers or reproductives to be singled out as a key driver, indicating that the whole microbial community contributes to these differences. Other colonies were experimentally exposed to antibiotics to alter their bacterial communities, and sampled shortly after treatment and weeks later after allowing for turnover of colony members. At those time points, bacterial communities of the two castes still differed across all antibiotic treatments; however, the caste ratio within colonies changed: after antibiotic disruption and turnover of individuals, new individuals were more likely to become reproductives than in undisturbed control colonies. Our results reveal that each caste has a distinct microbiome; whether the social context affects the microbiota, or whether microbes contribute to modulating the phenotype of individuals, remains to be determined.

## 1 INTRODUCTION

Division of labour, that is, the performance of different tasks by different individuals, is a central feature of social animals, like many hymenopteran insects where members of a colony are genetically related (Duarte et al., 2011; Rueffler et al., 2012). Colonies typically consist of a reproductive caste and other morphologically distinct castes that perform different functions for the colony's benefit (Fjerdingstad & Crozier, 2006). Functional specialization leads to increased efficiency and colony success, explaining why division of labour has repeatedly evolved in many taxa other than social insects in which individuals form groups of close kin (Simpson, 2012). These include several species of parasitic trematodes, in which an infective larva multiplies asexually within a snail intermediate host to form a colony of clonal individuals called rediae (Hechinger et al., 2011; Poulin et al., 2019; Whyte, 2021). In species with division of labour, rediae come as two distinct morphs. Large reproductive rediae have relatively small mouthparts and are mostly immobile; they produce dispersal stages known as cercariae that leave the snail to seek the next host in the parasite's life cycle. In contrast, “soldier” rediae are orders of magnitude smaller than reproductives and are incapable of producing cercariae. However, they have relatively large mouthparts, are highly mobile and aggressively defend the colony against other trematode competitors by attaching to them and killing them using their mouthparts (Hechinger et al., 2011; Leung & Poulin, 2011).

At the colony level, several external factors can shift the caste ratio to ensure adaptive responses to changing conditions. Since individual rediae have a short lifespan, there is a constant turnover of individuals within the colony. In the well‐studied trematode *Philophthalmus attenuatus*, colony composition responds within weeks to attempts by a competing trematode species to establish in their snail host by becoming increasingly soldier‐biased (Lagrue et al., 2018; Lloyd & Poulin, 2012). Similarly, *P. attenuatus* colonies also shift their caste ratio over just a few weeks in situations that compromise host survival, such as after their snail host is placed in conditions simulating ocean acidification (Guilloteau et al., 2016), or after a hole is drilled in the shell of their host (MacLeod et al., 2018).

These seemingly adaptive colony‐level responses do not explain why, at the individual level, a redia develops into a soldier instead of a reproductive, or vice versa. Because all colony members are clones, differential gene expression is a likely proximate mechanism. In social insects, for example, epigenetic processes are often invoked to explain how genetically similar individuals develop into phenotypically distinct castes (Oldroyd & Yagound, 2021; Pereboom et al., 2005; Scharf et al., 2003; Weiner & Toth, 2012). However, an individual's phenotype arises from interactions between not only its genome and the environment, but also the combined DNA of its resident microorganisms. There is growing evidence that an individual's microbiome, or the microbial community it harbours, can influence its development and eventual phenotype (Diaz Heijtz et al., 2011; Feldhaar, 2011). Among conspecific animals, distinct morphotypes or life history strategies are often associated with distinct endosymbiotic microbial communities (Leclair et al., 2016; Strunov et al., 2022; Takacs‐Vesbach et al., 2016). In social insects, members of different castes within the same colony often harbour different microbial symbionts (Kapheim et al., 2015; Koto et al., 2020; Poulsen et al., 2014), though whether the acquisition of a particular microbial community precedes or follows the developmental adoption of a particular caste remains unclear.

In social trematodes, could symbiotic microbes play a role in determining whether a redia adopts one caste over the other? Can certain components of the microbiome bias the development of their trematode carriers toward a particular strategy, for example, can vertically transmitted symbionts favour adoption of the reproductive caste? Does each caste have its unique microbial signature? These are questions that remain unanswered. Trematodes can harbour bacterial communities that are distinct from those of their host or the external environment, and that vary among individuals (Jorge et al., 2020). Several of these symbiotic bacteria are likely to be transmitted vertically through the parasite's life cycle (Jorge et al., 2020; Vaughan et al., 2012). This transmission route would not be available to bacteria within soldier rediae: as they do not produce dispersal stages, soldiers represent dead‐ends for their symbionts. Natural selection could therefore favour vertically‐transmitted bacteria capable of modulating the developmental route taken by the redia they occupy, resulting in individuals with and without these bacteria joining different castes and harbouring distinct bacterial communities.

Rediae of the social trematode *P. attenuatus* possess bacterial communities distinct from those of their hosts, with bacterial taxa unique to the parasite (Jorge, Dheilly, et al., 2022). Here, we test the hypotheses that (i) the much larger reproductive rediae harbour more diverse bacterial communities than soldiers; (ii) reproductives and soldiers harbour distinct bacterial communities in terms of their overall composition, even if neither caste is characterized by some unique taxa not shared with the other caste; and (iii) disrupting the bacterial communities in a redial colony will alter the subsequent development of new individuals into either soldiers or reproductives. We first tackled these hypotheses by characterizing and comparing the bacterial communities of reproductive and soldier rediae from the same colonies, that is, clones from the same snail host but belonging to different castes. Second, we experimentally disrupted the bacterial communities of redial colonies within their host, and monitored the subsequent changes in both the caste ratio of each colony and the bacterial community composition of members of the two castes. Assembling distinct microbiomes from scratch within microbe‐free rediae is impossible at present; however, disrupting existing microbial communities with antibiotics remains a powerful tool to demonstrate their association with redial phenotypes. Our findings show that different castes have distinct microbial communities, and that these differences are resistant to antibiotic treatment. Furthermore, the observed changes in caste ratio following antibiotic treatments support the hypothesis that microbes contribute to caste development. Our results suggest that microbes could contribute to colony structure and functioning in social trematodes.

## 2 MATERIALS AND METHODS

### 2.1 Study design and sample collection

Our model species is the trematode *Philophthalmus attenuatus* (Philophthalmidae). Adult worms live under the nictitating membrane in the eyes of sea gulls (*Larus* spp.), where they reproduce sexually. Their eggs accumulate in the bird's tears and end up on intertidal sediment. Miracidia hatched from those eggs seek and infect the snail *Zeacumantus subcarinatus*, which is the parasite's intermediate host. Within a snail, a single larva issued from one egg will undergo several rounds of asexual multiplication to form a clonal colony of rediae, including reproductive rediae that produce cercariae. After exiting the snail host, cercariae attach to and encyst (as metacercariae) on hard substrates, preferentially the outside surfaces of gastropod shells or crustacean exoskeletons. There, they await ingestion by a gull, after which they will excyst in the bird's gut and migrate to its eyes.

To test the prediction that particular microbes are associated with particular *P. attenuatus* phenotypic castes, we first determined the bacterial community composition of the two different castes within and across different snail hosts collected in the field (hereafter “natural” colonies). Then, we experimentally manipulated the bacterial communities of *P. attenuatus* colonies by exposing their snail host to different antibiotics (hereafter “experimental” colonies) to test whether particular microbes can bias redial development toward a particular caste, and quantified the effects on both colony size and the soldiers‐to‐reproductives ratio.

We targeted the bacterial kingdom as the microbial community of interest due to its wider characterization, and the high expected abundance of bacteria within trematode microbiota. Sample collection and the antibiotic treatment experiment followed the procedures of Jorge, Froissard, et al. (2022). In brief, >1500 snails, *Zeacumantus subcarinatus*, were collected from the intertidal zone in Lower Portobello Bay, South Island, New Zealand (45.83° S, 170.67° E) and screened in the laboratory (short‐term incubation at 25°C to induce release of cercariae) to identify individuals infected by *P. attenuatus*. All samples from the two parasite castes were isolated from snail tissue in sterile conditions. To achieve this, all dissections were conducted in a sterile laminar flow cabinet, and between each sample tools were cleaned with bleach, and sterilized with ethanol and burning flame. Snails were scrubbed with sterile interdental brush in 99% EtOH, and rinsed thoroughly in heat‐sterilized PBS prior to dissections. For natural colonies, two sets of three individual reproductives, and two sets of three individual soldiers from the same snail were isolated from 15 different snails, within 2 days following field collection. Experimental colonies consisted of four groups exposed to different antibiotic solutions (penicillin G potassium salt [5 g/L, Sigma P8721] targeting Gram‐positive bacteria; colistin sulphate salt [25 mg/L, Sigma C4461] targeting Gram‐negative bacteria; Gentamicin [50 mg/L, Sigma G3632] targeting both Gram‐positive and Gram‐negative bacteria and *Mycoplasma*; and a combination of penicillin and colistin [2.5 g/L and 12.5 mg/L, respectively]), and a fifth control group not exposed to antibiotics. Snails were exposed to antibiotics (or control seawater) for 3 days, then maintained in aerated seawater in a communal tank and fed ad libitum with thoroughly rinsed sea lettuce (*Ulva* spp.) from the collection site for 74 days. After the 3‐day exposure and at the end of the experiment, from each of the five groups, one set of three reproductives and one set of three soldiers from the same snail were isolated from each of 10 different snails per group and used for microbiome analysis. Full colonies occupying each of these 10 snail hosts per group were then censused to obtain the total number of rediae per colony, the number of individuals of each caste, and the soldiers‐to‐reproductives ratio. The reliability of using antibiotics to manipulate bacterial communities in this study system has been discussed elsewhere (Jorge, Froissard, et al., 2022). The corresponding study metadata is available in Table S1.

### 2.2 Sample processing, sequencing and bioinformatics

The data analysed in this study comprised the two data sets “natural” and “experimental” from which sequencing libraries were independently processed. Bacterial genomic DNA from each individual of the two separate castes, and from their “washing” (surface microbiota detached from the parasites by repeatedly pipetting them up and down, in PBS) was extracted using the DNeasy PowerSoil Pro Kit (Qiagen), with modifications recommended for cells difficult to lyse by the Earth Microbiome Project DNA Extraction Protocol (Marotz et al., 2017). ZymoBIOMICS microbial community standards samples (MCS), and reagent‐only samples were also included at both extraction and amplification steps. Metabarcoding libraries targeting the V4 hypervariable region of the prokaryotic bacterial 16S SSU rRNA gene were prepared as described in Jorge et al. (2020). Each barcoded libraries pool was sequenced on an Illumina MiSeq platform using the V2 reagent cartridge (250 bp, paired‐end) through the Otago Genomics & Bioinformatics Facility (New Zealand).

Raw demultiplexed reads were trimmed to remove adapters and primers using the plugin cutadapt (Martin, 2011) implemented in the QIIME2 software package (qiime2‐2020.2‐py36‐linux, Bolyen et al., 2019), and quality filtered using the dada2 plugin (Callahan et al., 2016). The resulting amplicon sequence variants (ASVs) tables were filtered to exclude non‐bacterial, mitochondrial, chloroplast, ASVs without a phylum assignment, contaminants (i.e., ASVs found in blanks and exclusive to the laboratory environment), ASVs found in the corresponding “washing”, and samples with low sequencing depth (i.e., frequency lower than 1000 reads and/or with <8 ASVs). Different taxonomic levels were assigned to the ASVs using the plugin feature‐classifier (Bokulich et al., 2018) against the Silva (138 release) 16S rRNA reference database (Quast et al., 2013) pretrained on the 515F/806R region (Pedregosa et al., 2011).

### 2.3 Statistical analysis

All statistical analyses were performed using R, version 4.1.3 (R Core Team, 2022), using mainly the R packages vegan version 2.6–2 (Oksanen et al., 2019) and phyloseq version 1.40.0 (McMurdie & Holmes, 2013) unless stated otherwise. Differences in diversity between castes (alpha diversity) were estimated using Faith's phylogenetic diversity, evenness and Shannon diversity metrics on rarefied data across several taxonomic groups, and assessed with Kruskal–Wallis tests. Community level differences (beta diversity) were estimated with the phylogeny‐based indices, unweighted Unifrac (Lozupone & Knight, 2005) and quantitative weighted UniFrac (Lozupone et al., 2007). Statistically significant differences between castes were determined with permutational ANOVA performed with adonis2() with 9999 permutations. Principal coordinates plots (PCoA) were created with plot_ordination() adding hulls as defined with find_hull() from the R package erictools version 0.0.0.9 (https://rdrr.io/github/elittmann/erictools/man/find_hull.html). Differential abundance of taxa between groups was modelled with the corncob package version 0.2.0 (Martin et al., 2020). The association between caste and microbial communities was modelled using a data‐driven adaptive test of GLMM‐MiRKAT (aGLMM‐MiRKAT) based of multiple ecological distances (Jaccard, Bray‐Curtis and UniFrac distances), from the R package GLMMMiRKAT version 1.2 (Koh, 2020). Microbial community colonization dynamics within the same colony was estimated by comparing ASV sharing and their respective proportion for each pair of samples between and within castes originating from the same host individual, based on the R script from Maqsood et al. (2019).

To determine if each parasite caste has its unique microbial signature, we compared estimated diversity and community indices, explored differential abundance and differential variability between castes, calculated as described above, focusing on the “natural” colonies data set. We further explored the variability of microbial community composition between reproductives and soldiers from the same parasite colony.

To test for a potential bias in parasite development due to microbial composition, we compared the bacterial diversity and community composition of the two castes within “experimental” colonies, and related the differences to changes in parasite colony size and soldiers‐to‐reproductives ratio.

## 3 RESULTS

### 3.1 Data overview

A total of 55 samples (27 soldiers, and 28 reproductives) were successfully isolated from the natural colonies, 94 samples (47 soldiers, and 47 reproductives) for the experimental colonies 3 days post‐treatment, and 100 samples (50 soldiers, and 50 reproductives) at the end of the experiment. After sequence and sample quality control filtering (see Methods), 35 (12 soldiers with a total of 199 ASVs, and 23 reproductives with a total of 695 ASVs), 70 (33 soldiers with a total of 550 ASVs, and 37 reproductives with a total of 1185 ASVs), and 75 (33 soldiers with a total of 574 ASVs, and 42 reproductives with a total of 909 ASVs) samples were included for the final natural colonies, 3‐day post‐treatment experimental colonies, and end‐of‐experiment colonies, respectively. The blank samples for the natural (*N* = 9), post‐treatment experimental (*N* = 6) and end‐of‐experiment (*N* = 6) colonies contained 85, 149, and 79 ASVs, respectively (43, 67 and 36 unique to the blanks). Quality control analyses based on the observed and expected MCS did not detect bias in extraction and amplification of the different bacteria composing the MCS (i.e., all eight bacteria were successfully amplified), or errors in sequencing (i.e., no sequence mismatches). Cross contamination was detected between microbial community standards samples (MCS) and blanks, but given that all features found in blanks were removed from the data sets there was no influence on the following analyses.

### 3.2 Does each caste have its unique microbial signature?

**FIGURE 1:**
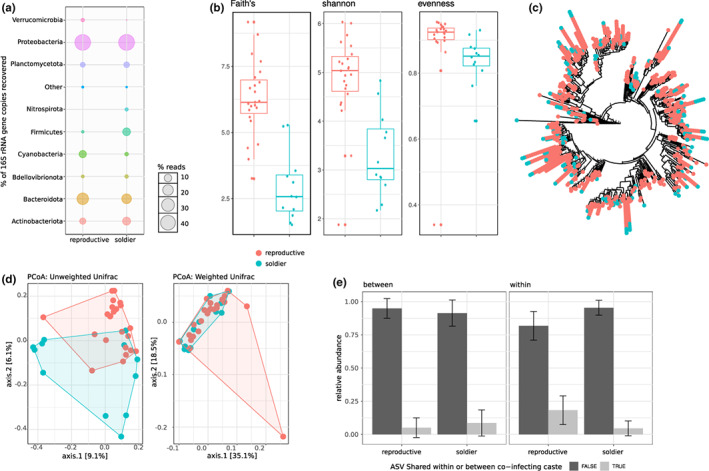
Each caste of the social trematode *Philophthalmus attenuatus* has its unique microbial community. (a) Relative abundance of bacterial phyla occurring in each of the two castes. “Other” represents all taxa whose relative abundances are <1% of the total abundance. (b) Alpha diversity of bacteria in the two castes as measured using Faith's phylogenetic, Shannon and evenness diversity indices at ASV‐level. (c) Phylogenetic tree showing the relationships among ASVs making up the bacterial communities in the two castes. Dots are shown when a taxon is observed in a given sample. (d) Principal coordinates analysis ordinations based on unweighted and weighted unifrac distance matrices, with hulls delimiting groups of samples from each caste. (e) Average relative abundance of ASVs (as a percentage) in samples that are shared within or between the two castes within the same snail host.

In natural colonies, the bacterial communities of both parasite castes were dominated by Proteobacteria (soldier = 47.43%, reproductive = 46.49%) and Bacteroidota (soldier = 17.16%, reproductive = 24.61%) (Figure 1a). However, apart from this similarity, the two castes presented significant differences in both diversity and community composition. The reproductive caste presented significantly higher community richness at the tested taxonomic levels from ASV to phylum, and higher evenness (the latter only estimated at ASV‐level; Figure 1b, Table S2). There were also overall differences in community composition between the two castes (Figures 1c–d, Table S3). At the lowest taxonomic level, the communities clustered separately by caste based on composition (unweighted unifrac: *p* = .001, and betadisper: *p* = .066), although not when considering the abundances of bacterial taxa (weighted unifrac: *p* = .093, and betadisper: *p* = .981). However, at all other taxonomic levels considered and for both unweighted and weighted unifrac estimates, the two castes presented significant differences, and also significant differences in homogeneity of dispersion within caste (Table S3). We also found a significant association between parasite caste and microbial composition when using aGLMM‐MiRKAT analysis for the five taxonomic levels tested (all cases, *p* < .001). These differences were not supported by changes in the relative abundance or variability of particular taxa. Only at phylum and class level was there support for a higher variability in the abundance of some taxa between the two castes, that is, Planctomycetota (accounting for approximately 5% of the community composition in each caste), Firmicutes (accounting for approximately 10% of the community composition in soldiers, and 1% in reproductives), and at class level Gammaproteobacteria (Proteobacteria). All three taxa were significantly more variable in their abundances in soldiers. The community composition of each caste was variable, with no taxon being shared by all individuals of the same caste, but also within the same host, with a very low proportion of taxa (ASVs) being shared whether within or between the two castes (Figure 1e). On average, the two castes share two bacterial taxa which accounted for 5.03% and 8.59% of the total reads in reproductives and soldiers, respectively. Within each caste, shared taxa represent a greater relative abundance in reproductives than soldiers (Figure 1e).

### 3.3 Can microbes bias parasite development toward one caste or the other?

**FIGURE 2:**
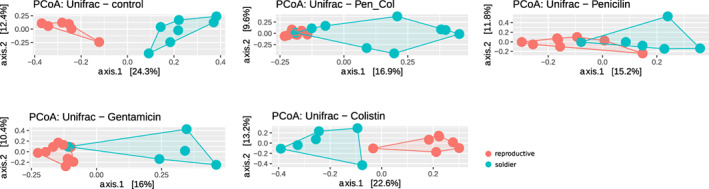
Each caste of the social trematode *Philophthalmus attenuatus* has its unique microbial community regardless of the antibiotic treatment. Principal coordinates analysis ordinations based on unweighted unifrac distance matrices, with hulls delimiting groups of samples from each caste, for all five treatments.

While we found that each caste harboured a distinct bacterial community, only three higher level taxa (phylum: gram‐negative Planctomycetota, gram‐positive Firmicutes; class: gram‐negative Gammaproteobacteria) differed significantly in variability of the abundance between the two castes, whereas no taxon differed between castes in actual abundance. In fact, differences between the two castes seem to be driven by overall unique community composition. To assess whether microbes bias parasite development toward one caste or the other, we experimentally manipulated the bacterial composition of colonies with broad‐spectrum antibiotics and monitored changes in the community composition, and in colony size and soldiers‐to‐reproductives ratio within each host. In these experimental colonies, within the same caste, we did not find support for significant changes in overall microbial community composition among the antibiotic treatments and the control group 3 days post‐exposure for most taxonomic levels (Table S4), which could in part be due to low sample size. There were detectable effects of antibiotics on the microbial communities of the parasite, however; no individual treated with gentamicin contained any ASV assigned to the family Mycoplasmataceae, whereas representatives of this family were commonly found in individuals from other treatments (see also Jorge, Froissard, et al., 2022). More importantly, when comparing between the two castes, the bacterial communities differed in terms of composition (unweighted Unifrac) among all the antibiotic treatments (Figure 2, Table S5). When considering abundance (weighted Unifrac), significant differences persisted between the two castes, except for colistin, penicillin, and at some taxonomic levels of the penicillin‐colistin treatment (Table S5). These differences in community composition were maintained until the end of the experiment (Table S6). PERMANOVA shows that only caste, and not the antibiotic treatment or their interaction, had an effect accounting for differences between the microbial communities (Table S7).

**FIGURE 3:**
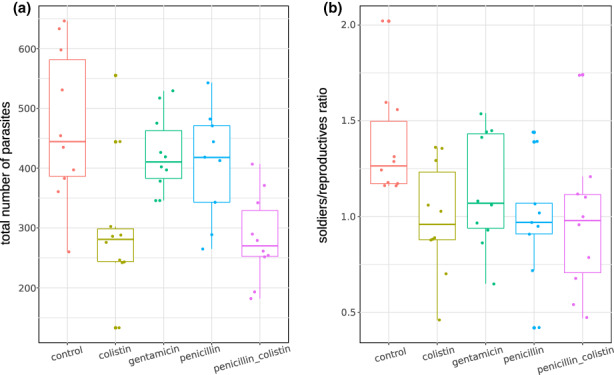
Antibiotic treatments lead to changes in parasite colonies. (a) Effect on colony size, the total number of individuals per colony, and (b) changes in the soldiers‐to‐reproductives ratio in colonies of the social trematode *Philophthalmus attenuatus*, shown here for each antibiotic treatment. Each point represents one colony.

Antibiotic exposure was associated with changes in the parasite colonies at the end of the experiment. There was evidence for an effect on colony size, that is, on the total number of parasites (Kruskal‐Wallis chi‐squared = 18.802, *df* = 4, *p* = .001), and for significant changes in the caste ratio (Kruskal‐Wallis chi‐squared = 10.713, df = 4, *p* = .030, Figure 3). Both the colistin and penicillin‐colistin treatments lead to a significant decrease in the colony size (Wilcoxon rank sum test; *p*‐adjusted = .022, and *p*‐adjusted = .008, respectively).

## 4 DISCUSSION

The mechanisms underpinning the differentiation of genetically identical individuals into phenotypically and functionally distinct castes remain poorly understood. Here, we show that members of two different castes in a social trematode harbour distinct microbiomes, and we further propose that the microbiomes of individuals might play a role in caste development. There is mounting evidence that an individual's microbiome can shape its phenotype (Diaz Heijtz et al., 2011; Feldhaar, 2011; Leclair et al., 2016; Strunov et al., 2022; Takacs‐Vesbach et al., 2016). On evolutionary timescales, microbiomes may even allow rapid adaptation (Alberdi et al., 2016) as well as influence animal speciation (Miller et al., 2021). In this study, we provide some evidence suggesting that they may also affect animal phenotype on developmental timescales. Following from work in social insects suggesting that members of different castes may harbour different microbial symbionts (Kapheim et al., 2015; Koto et al., 2020; Poulsen et al., 2014), we tested three hypotheses regarding the association of symbiotic bacteria with caste differentiation in the social trematode *Philophthalmus attenuatus*, and found support for all three.

Firstly, we found that reproductive rediae harbour more diverse bacterial communities than soldiers, regardless of the diversity index used or the taxonomic level considered. Reproductives and soldiers are clones, with the same genotype and developing in the same within‐host environment. Differences in their microbiomes may simply be a consequence of the huge size difference between members of the two castes, as space limitation may constrain overall bacterial diversity (Reese & Dunn, 2018). Within each caste, however, community composition varied among individuals, with no taxon shared by all members of the same caste, even individuals from the same colony (i.e., from the same snail host). This may be explained by both imperfect vertical transmission and a founder effect during the asexual production of rediae, with each individual not necessarily inheriting the full complement of bacterial taxa characteristic of its caste. The proportion of shared taxa was lower among soldiers than among reproductives, which may again be a consequence of the smaller size of soldiers. However, the greater variability and lower consistency of bacterial communities within the soldier caste may also reflect the fact that they are dead‐ends for vertical transmission, weakening any pressure on microbes to achieve high rates of initial colonization of these rediae.

More importantly, our second hypothesis, that is, reproductives and soldiers harbour distinct bacterial communities, in terms of their composition, was also supported. This is generally true at all taxonomic levels considered, and whether or not the relative abundances of different bacterial taxa were taken into account. One possibility is that differences in anti‐bacterial immune response abilities between soldiers and reproductives result in differences in microbiome composition. Indeed, a better ability to control microbiome composition in reproductives might explain why a greater number of taxa are shared among reproductives than soldiers. The result is also consistent with the idea that symbiotic microbes are associated with the development of the rediae they inhabit into one caste or the other. Bacteria that are exclusively, or mostly, vertically transmitted would benefit the most from nudging redial development toward the reproductive caste. Two bacterial phyla, gram‐negative Planctomycetota, and gram‐positive Firmicutes, and one class, the gram‐negative Gammaproteobacteria (Proteobacteria), showed differential abundance between the two castes. Some of these, such as Planctomycetota, are vertically transmitted in other invertebrate species in which they occur as symbionts (Björk et al., 2019). However, whether members of this bacterial taxon are particularly influential relative to other bacteria in driving redial development into one or the other caste remains to be determined. Admittedly, the lack of consistency in bacterial community composition among individual rediae of the same caste weakens the support for our hypothesis. No bacterial taxon is shared by all members of the same caste. Therefore, influences on redial development may originate from a range of bacteria that lie along a gradient from beneficial to detrimental interacting with the rediae, with redial phenotype being the result of complex and multifactorial interactions between multiple bacterial taxa.

The differences in the composition of bacterial communities between members of the two castes in natural colonies of *P. attenuatus* provides only correlative evidence of an association between microbiomes and trematode castes. Variation in bacterial community composition between the two castes may either play a causal role early in development in determining whether the rediae adopt one caste over another, or they may arise later as a consequence of the rediae having adopted a particular caste. Experimental disruption of microbial communities is a powerful complementary approach to demonstrate causality (e.g., Martinson et al., 2020). If microbiome composition results strictly from differences in body size between the two castes, or from inherited differences in their antibacterial response abilities, then experimental microbiome disruption should not affect the caste ratio. On the contrary, if the microbiome contributes to driving caste development, then disrupting the microbiome should affect caste ratio. We used antibiotic treatments to disrupt the microbial communities of rediae within their host to address our third hypothesis that such a disruption should alter the colony's caste ratio. Exposure to antibiotics caused a reduction in *P. attenuatus* colony size in two cases (colistin and penicillin‐colistin) compared to colonies in the control group, and a general decrease in the soldiers‐to‐reproductives caste ratio. Thus, after having their bacterial communities disrupted by antibiotics and allowing time for a turnover of individuals within the colony, new rediae were more likely to become reproductives than in undisturbed control colonies. This supports the hypothesis that microbiome composition may affect redial development and which caste they join.

After the initial exposure to antibiotics, the bacterial communities of rediae changed quickly, but kept evolving over time toward a new and possibly stable state by the end of the experiment (see Jorge, Froissard, et al., 2022). Niches left vacant after the initial disruption were filled by bacterial taxa that survived the treatment or colonized from outside the rediae. Interestingly, 3 days post‐treatment and at the end of the experiment the bacterial communities of the two castes differed in terms of composition (unweighted Unifrac analyses) across all the antibiotic treatments. This is a striking result, whichever way the causal arrow between microbiome and phenotype is pointing. Knocking out certain taxa did not subsequently cause the bacterial communities of both castes to converge. Although all rediae, both those pretreatment and those developing post‐treatment, are clones originating from the same germ cells, perhaps the factors shaping bacterial communities in the two castes are too distinct for the differences in bacterial community composition to disappear even as new colony members replaced deceased ones. Again, the result is consistent with the composition of the bacterial community influencing which caste a redia will develop into.

We must address three alternative explanations associated with our protocol. These alternatives are largely inevitable given that we are working with a Russian doll system, that is, studying the microbiome within parasites, themselves within a host. First, exposure of rediae within their snail host to antibiotics also resulted in changes to the snail's own microbial communities (Jorge, Froissard, et al., 2022). Snail microbiomes are known to influence trematode parasites and how they interact with their host (Le Clec'h et al., 2022). It is therefore possible that certain bacteria affecting redial development into either soldiers or reproductives belong to the snail's microbial communities rather than to the parasite's microbial communities. However, this seems less likely as the caste into which a redia develops should have much greater fitness consequences for its bacteria than for those of the snail host, leading to greater selective pressures on bacteria within the trematode to evolve the ability to influence their development. Second, antibiotic exposure may have induced stress in the snail host. Changes in the caste ratio of *P. attenuatus* colonies following exposure of their snail host to other stressors have been previously documented (Guilloteau et al., 2016; MacLeod et al., 2018). We cannot rule out that our findings result, at least in part, from a response to host stress. Third, antibiotic exposure may have directly killed rediae of one caste more than the other, or bacteria essential to their survival. This seems unlikely, however, as there was no difference in the total number of rediae per colony 3 days after exposure among all treatments and controls (results not shown).

The present results provide further support for our growing understanding of the association between parasite microbiomes and parasite biology and host–parasite interactions (Dheilly et al., 2015, 2019). Furthermore, they reveal that as suggested in some social insects (Kapheim et al., 2015; Koto et al., 2020; Poulsen et al., 2014), the social structure and division of labour within trematode colonies correlate with the diversity and nature of the microbes they harbour, and provide clues toward determining whether microbes modulate the parasite's phenotype. Microbiomes may also account for some of the intraspecific variation in colony structure on larger spatial scales. Indeed, since the bacterial communities of the social trematode *P. attenuatus* vary among geographic locations (Jorge, Dheilly, et al., 2022), the role of microbes in shaping redial development may explain the earlier observation that the intrinsic caste ratios of *P. attenuatus* colonies also vary among the same geographic locations (Lloyd & Poulin, 2014). Our findings suggest that the evolution of sociality in trematodes, and possibly other animal taxa, should perhaps be considered as the outcome of diffuse coevolution between social animals and the influential symbiotic microbes they carry.

## AUTHOR CONTRIBUTIONS

Robert Poulin, Fátima Jorge and Nolwenn Dheilly conceived and designed the study. Fátima Jorge and Céline Froissard conducted the field sampling and the laboratory work. Fátima Jorge conducted all genetic, bioinformatic and statistical analyses, with input from Céline Froissard, Nolwenn Dheilly and Robert Poulin. Fátima Jorge and Robert Poulin cowrote the manuscript, with input from Nolwenn Dheilly and Céline Froissard.

## CONFLICT OF INTEREST

The authors have no competing interests.

## Supporting information


Appendix S1

## Data Availability

Sequences for all of the samples were submitted to and deposited in the NCBI sequence read archive (SRA) under accession reference PRJNA707308 and PRJNA786706.
